# A New Visualization for Probabilistic Situations Containing Two Binary Events: The Frequency Net

**DOI:** 10.3389/fpsyg.2020.00750

**Published:** 2020-05-26

**Authors:** Karin Binder, Stefan Krauss, Patrick Wiesner

**Affiliations:** Mathematics Education, Faculty of Mathematics, University of Regensburg, Regensburg, Germany

**Keywords:** frequency net, natural frequencies, conditional probabilities, joint probabilities, Bayesian reasoning

## Abstract

In teaching statistics in secondary schools and at university, two visualizations are primarily used when situations with two dichotomous characteristics are represented: 2 × 2 tables and tree diagrams. Both visualizations can be depicted either with probabilities or with frequencies. Visualizations with frequencies have been shown to help students significantly more in Bayesian reasoning problems than probability visualizations do. Because tree diagrams or double-trees (which are largely unknown in school) are node-branch structures, these two visualizations (in contrast to the 2 × 2 table) can even simultaneously display probabilities on branches *and* frequencies inside the nodes. This is a teaching advantage as it allows the frequency concept to be used to better understand probabilities. However, 2 × 2 tables and (double-)trees have a decisive disadvantage: While *joint probabilities* [e.g., P(A∩B)] are represented in 2 × 2 tables but no *conditional probabilities* [e.g., P(A|B)], it is exactly the other way around with (double-)trees. Therefore, a visualization that is equally suitable for the representation of joint probabilities *and* conditional probabilities is desirable. In this article, we present a new visualization*—the frequency net—*in which all absolute frequencies and all types of probabilities can be depicted. In addition to a detailed theoretical analysis of the frequency net, we report the results of a study with 249 university students that shows that “net diagrams” can improve reasoning without previous instruction to a similar extent as 2 × 2 tables and double-trees. Regarding questions about conditional probabilities, frequency visualizations (2 × 2 table, double-tree, or net diagram with absolute frequencies) are consistently superior to probability visualizations, and the frequency net performs as well as the frequency double-tree. Only the 2 × 2 table with frequencies—the one visualization that participants were already familiar with—led to higher performance rates. If, on the other hand, a question about a joint probability had to be answered, all implemented visualizations clearly supported participants’ performance, but no uniform format effect becomes visible. Here, participants reached the highest performance in the versions with probability 2 × 2 tables and probability net diagrams. Furthermore, after conducting a detailed error analysis, we report interesting error shifts between the two information formats and the different visualizations and give recommendations for teaching probability.

## Introduction

Experimental cognitive psychology research on the effects of natural frequencies and visualizations focuses primarily on *conditional probabilities*, especially on Bayesian tasks like the famous mammography problem and similar cognitive illusions like the Monty Hall problem (Kahneman et al., 1982; Gigerenzer and Hoffrage, 1995; Goodie and Fantino, 1996; Hoffrage et al., 2000; Krauss and Wang, 2003; Barbey and Sloman, 2007; Spiegelhalter et al., 2011; Baratgin, 2015; Operskalski and Barbey, 2016; McDowell and Jacobs, 2017).

However, conditional probability tasks, and especially Bayesian tasks are only one aspect of teaching probability at secondary schools and university. Tasks on *joint probabilities* also play an important role in stochastic education, as they contribute significantly to the general understanding of probabilities (see, e.g., Pfannkuch and Budgett, 2017). In this article, we seek to broaden the field of natural frequencies and visualizations in Bayesian reasoning to questions about joint probabilities and to that end present a new visualization that is equally suitable for both types of probabilities.

In the teaching of statistics at secondary school and university level, two visualizations are primarily used when situations with two dichotomous characteristics are represented: 2 × 2 tables and tree diagrams. Both visualizations can be depicted with probabilities or with frequencies. Visualizations with frequencies have been shown to help students significantly more than probability visualizations in Bayesian reasoning problems (Binder et al., 2015, 2018). Tree diagrams and their extensions to double-trees can even display both information formats simultaneously, which is an advantage from a pedagogical point of view.

However, 2 × 2 tables and (double-)trees each have a decisive disadvantage with respect to the probability representation: While in 2 × 2 tables, aside from marginal probabilities, *only joint probabilities* [e.g., P(A∩B)] are represented but no conditional probabilities [e.g., P(A|B)], (double-)trees present *conditional probabilities* but no joint probabilities. Although it is possible to see joint probabilities in the double-tree with frequencies by skipping a level and reading “160 of 10,000,” there is no branch provided to display the corresponding joint probabilities directly, which has disadvantages from an educational point of view (the same holds true for 2 × 2 tables).

In this article we present a new visualization—the frequency net (also a node-branch structure)—in which *all* frequencies as well as *all* probabilities can be depicted simultaneously. In section “The Frequency Net and Net Diagrams” a detailed theoretical analysis of this new visualization is presented. Furthermore, we will report results of an empirical study on this visualization, conducted with 249 university students, in which we systematically varied the information format (probabilities vs. frequencies) and the visualization (no visualization, 2 × 2 table, double-tree, or frequency net) of the task. In addition to the typical questions for conditional probabilities, we also asked joint probability questions. Finally, a systematic analysis of the typical errors that occurred is presented—separately for information format, visualization and inference type.

## Visualizations of Statistical Information

### Conditional Probabilities and Bayesian Reasoning

Many professionals, like medical doctors and judges in court have to make important decisions based on statistical information. Often, Bayesian inferences are necessary for such decision-making processes, for example when a radiologist has to assess and communicate the statistical meaning of, for instance, a positive mammography screening. Many empirical studies have documented faulty inferences and even cognitive illusions among professionals of various disciplines, like physicians (Hoffrage and Gigerenzer, 1998; Garcia-Retamero and Hoffrage, 2013), those in the legal profession (Hoffrage et al., 2000), and managers (Hoffrage et al., 2015a), as well as secondary or university students (Ellis et al., 2014; Binder et al., 2015; Böcherer-Linder and Eichler, 2019).

Consider, for instance, the mammography problem, in which the prevalence of the disease has to be linked with the sensitivity and the false-positive rate for a mammogram in order to determine the probability that a woman with a positive mammogram actually has breast cancer (adapted from Eddy, 1982; see also Gigerenzer and Hoffrage, 1995; Siegrist and Keller, 2011; Micallef et al., 2012; Garcia-Retamero and Hoffrage, 2013; the numbers given below were adjusted in such a way that the positive predictive value corresponds to the one from the current German mammography screening report, Kooperationsgemeinschaft Mammographie, 2018).

#### Mammography Problem – Probability Format

The probability of breast cancer is 2% for a woman of a particular age group who participates in a routine screening. If a woman who participates in a routine screening has breast cancer, the probability is 80% that she will have a positive mammogram. If a woman who participates in a routine screening does not have breast cancer, the probability is 10% that she will have a false-positive mammogram.

What is the probability that a woman of this age group who participates in a routine screening and has a positive mammogram actually has breast cancer?

The correct solution can be determined using Bayes’ formula and is about 14%. However, most people in reality estimate such (*a posteriori*) probabilities to be much higher (Eddy, 1982; Hoffrage and Gigerenzer, 1998). In the last 25 year, to help prevent that kind of dangerous misjudgment, research has intensively examined the concept of natural frequencies in Bayesian reasoning problems, both theoretically and empirically (Gigerenzer and Hoffrage, 1995; Hoffrage and Gigerenzer, 1998; McDowell and Jacobs, 2017; McDowell et al., 2018). These studies have shown that many more people are able to answer this type of question if all statistical information is presented using natural frequencies rather than confusing probabilities:

#### Mammography Problem – Natural Frequency Format

200 out of 10,000 women of a particular age group who participate in a routine screening have breast cancer. 160 out of 200 women who participate in a routine screening and have breast cancer will have a positive mammogram. 980 out of 9,800 women who participate in a routine screening and have no breast cancer will have a false-positive mammogram.

How many of the women of this age group who participate in a routine screening and receive positive mammograms actually have breast cancer?

This mode of representation makes it possible to imagine concrete persons, the nested-set relations get transparent, and thus the solution algorithm becomes simpler. Now it is easy to see that 160 + 980 women receive positive mammograms and only 160 out of these 1,140 women actually have breast cancer. A recent meta-analysis by McDowell and Jacobs (2017) summarized 35 studies that implemented natural frequencies and found an average performance in natural frequency versions of Bayesian reasoning problems of about 24%, compared to only 4% in studies that used probability versions (for details see McDowell and Jacobs, 2017).

Another strategy for improving Bayesian reasoning is using visualizations such as *2* × *2 tables* (Steckelberg et al., 2004; Binder et al., 2015), *tree diagrams* (Sedlmeier and Gigerenzer, 2001; Yamagishi, 2003; Steckelberg et al., 2004; Binder et al., 2015; Budgett et al., 2016; Reani et al., 2018), *double-trees* (Wassner, 2004; Khan et al., 2015; Böcherer-Linder and Eichler, 2019), *Euler diagrams* (Sloman et al., 2003; Micallef et al., 2012; Sirota et al., 2014; Reani et al., 2018), *roulette-wheel diagrams* (Yamagishi, 2003; Brase, 2014), *frequency grids* (Cosmides and Tooby, 1996; Sedlmeier and Gigerenzer, 2001; Garcia-Retamero et al., 2015), *Eikosograms* (sometimes also called *unit squares* or *mosaic plots*; e.g., Oldford and Cherry, 2006; Böcherer-Linder and Eichler, 2017; Pfannkuch and Budgett, 2017; Talboy and Schneider, 2017), or *icon arrays* (Zikmund-Fisher et al., 2014; Brase, 2008, 2014; Reani et al., 2018). Since the visualization of statistical information is as successful as the natural frequency strategy (McDowell and Jacobs, 2017), there have also been efforts in recent times to develop new visualizations with specific advantages, such as the *dot diagram* (which is a hybrid visualization of a 2 × 2 table, an Euler diagram, and an icon array; Wu et al., 2017) the *turtleback diagram* (Yan and Davis, 2018), or interactive diagrams like *pachinkograms* (Budgett and Pfannkuch, 2019; Starns et al., 2019). For an overview of typical visualizations for situations with two dichotomous characteristics, see Spiegelhalter et al. (2011), or Binder et al. (2015), and for a classification of typical visualizations used for branching, nested-set relation, or frequency, see Khan et al. (2015).

**FIGURE 1:**
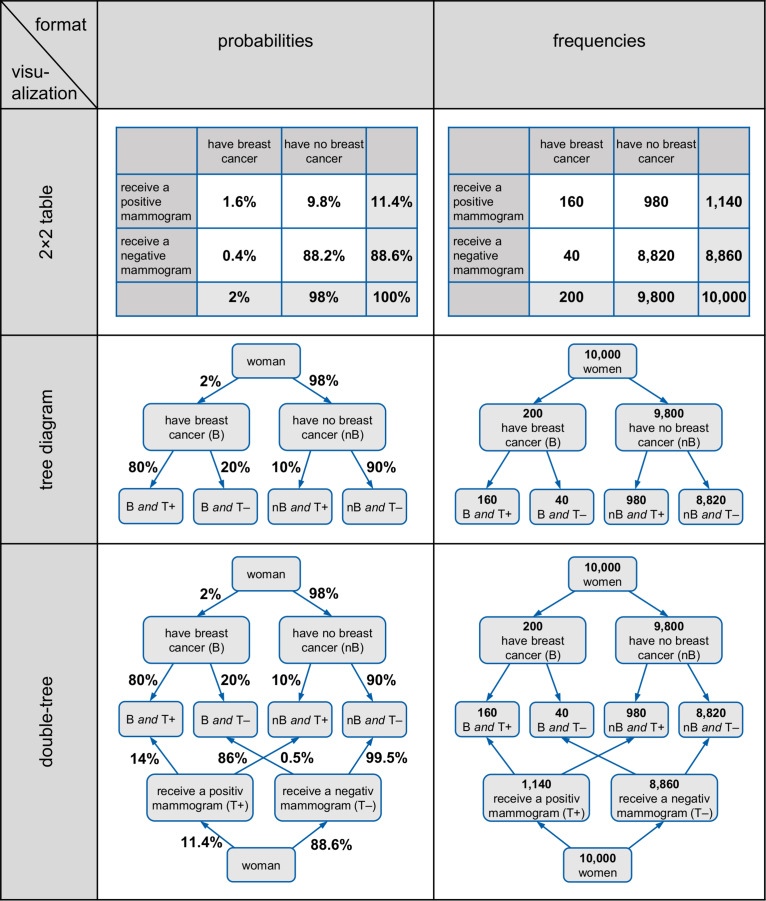
2 × 2 tables, tree diagrams, and double-trees (left in probabilities, right in frequencies) for the mammography problem.

Note that 2 × 2 tables, tree diagrams, and double-trees all have the advantage that they can be constructed easily with paper and pencil by teachers or students. In contrast, most of the other diagrams mentioned above are complicated to produce, which is especially problematic when base rates are extreme (as in typical medical Bayesian reasoning problems). Area-proportional Euler diagrams, for instance, are unsuitable for teaching because drawing such illustrations is geometrically difficult. In the Eikosogram, areas can become so small that they can almost no longer be effectively represented in the diagram (if the base rate is very small). Similarly, the icon array is based on small symbols instead of geometrical areas, and thus many symbols have to be produced in the case of small or unmanageable proportions (such as 0.1%), entailing an enormous amount of effort to draw, for instance, 1,000 or even in some cases 10,000 small icons. Therefore the focus of this article is on 2 × 2 tables, tree diagrams, and double-trees, which are displayed in Figure 1.

Furthermore, these three visualizations usually display the statistical information explicitly as numbers. In these visualizations, the statistical information can be expressed either as probabilities or as absolute frequencies (see, e.g., Figure 1) but only in (double-)trees can both formats be displayed *simultaneously*.

However, from an educator’s point of view, it would be helpful if a visualization could display both absolute frequencies and probabilities simultaneously because this would allow one to switch representations instantly and to see the meaning of marginal probabilities, conditional probabilities, or joint probabilities in terms of intuitive absolute frequencies that could be combined to natural frequencies (e.g., “160 out of 200”). Yet only in *node-branch structures* like tree diagrams and double-trees—but not in 2 × 2 tables—can absolute frequencies and probabilities be displayed *at the same time* (see, e.g., “branching,” Khan et al., 2015). Note that these visualizations are especially helpful when they contain absolute frequencies rather than probabilities (e.g., Binder et al., 2015; Bruckmaier et al., 2019).

### From Bayesian Reasoning to Other Statistical Judgments: Teaching Probability in Secondary School and University

In teaching probability and statistics at secondary school level, Bayesian tasks are only one of a number of probability tasks covered. In fact, there are 16 different probabilities in a situation with two dichotomous events (A and B): Four marginal probabilities [P(A), P($\bar{\text{A}}$), P(B), P($\bar{\text{B}}$)], four joint probabilities [P(A∩B), P(A∩$\bar{\text{B}}$), P($\bar{\text{A}}$∩B), P($\bar{\text{A}}$∩$\bar{\text{B}}$)], and eight conditional probabilities [P(A|B), P($\bar{\text{A}}$|B), P(A|$\bar{\text{B}}$), P($\bar{\text{A}}$|$\bar{\text{B}}$), P(B|A), P($\bar{\text{B}}$|A), P(B|$\bar{\text{A}}$), P($\bar{\text{B}}$|$\bar{\text{A}}$)]. Thus far, research on the effect of natural frequencies and visualizations predominantly focuses on the notoriously difficult Bayesian conditional probabilities (for exceptions, see Böcherer-Linder and Eichler, 2017; Bruckmaier et al., 2019) due to their impact for important real-world decisions in many domains (see, e.g., Hoffrage et al., 2000; Operskalski and Barbey, 2016).

However, judgment errors with severe consequences can also occur in connection with joint probabilities, for example in association with the difficult concept of independence of events such as occurred in the famous trial of Sally Clark (see, e.g., Schneps and Colmez, 2013; Barker, 2017; Jessop, 2018). In this trial, Sally Clark was charged with murdering her two infant sons, who had actually died of sudden infant death syndrome (SIDS). The court expert Roy Meadow made two probabilistic judgment errors here: (1) The court committed the typical “prosecutor’s fallacy” (Hill, 2004), which again is based on misinterpretation of conditional probabilities; and (2) Meadows’ calculation was based on the assumption that two SIDS within a family are stochastically independent, which is not the case. Thus, because of their mathematical value as well as because of their practical relevance, the typical (Bayesian) inverted conditional probabilities should be examined, but—importantly—also joint probabilities, especially when it comes to the visualization of these probabilities.

Table 1 shows four potential advantageous features of visualizations in situations with two dichotomous events: (1) The possibility to display all four joint probabilities directly, (2) the possibility to display all eight conditional probabilities, (3) the possibility to display probabilities and frequencies simultaneously (then it is possible to understand *probabilities* with the help of *frequencies*), and (4) the possibility for both reading directions to be represented at the same time. Therefore, Table 1 shows the suitability of 2 × 2 tables, trees, and double-trees for visualizing those 16 probabilities that can occur in situations with two dichotomous events [besides P(Ω) and P(Ø)]. This results in a disadvantage for teaching mathematics: Either the students learn to always select the appropriate visualization for each task, or they have to accept the fact that they sometimes first have to perform an extra calculation before the visualization can be completed (for a detailed explanation, see Binder et al., under review). If, for example, only joint probabilities are given in a task, these probabilities cannot be written directly into the double-tree—because in double-trees, no branch is available for depicting joint probabilities. In this case, the joint probabilities must be converted in a previous calculation into conditional probabilities, which can then be displayed in the (double-)tree.

**TABLE 1:** Advantages and disadvantages of 2 × 2 tables, trees, double-trees, and net diagrams.

Advantage	2 × 2 table	Tree diagram	Double-tree	Net diagram
All joint probabilities can be displayed directly	✓			✓
All conditional probabilities can be displayed directly		(Only 4 out of 8)	✓	✓
Probabilities and frequencies can be presented simultaneously		✓	✓	✓
Both “reading directions” are equally evident	✓		✓	✓

Furthermore, as mentioned above, the double-tree as a node-branch structure has one feature that might be an advantage for *teaching—*compared to the 2 × 2 table*—*because it can represent probabilities as well as frequencies, including their mutual relations at the same time. In contrast to what one sees in “basic” tree diagrams, both reading directions are simultaneously evident in double-trees. However, even the advantageous double-tree has three disadvantages:

• *Missing joint probabilities:* There are no branches on which the (four) joint probabilities can be directly depicted. If such branches are added, the diagram becomes cumbersome.
• *Crossing branches:* Two branches overlap in the lower part of the double-tree. This may be problematic for learners, since it carries the risk of confusing the conditional probabilities that are positioned on the two crossing branches.
• *Doubled node:* One of the nodes of the double-tree appears twice—namely the one that represents the total sample (e.g., 10,000 women).

### The Frequency Net and Net Diagrams

**FIGURE 2:**
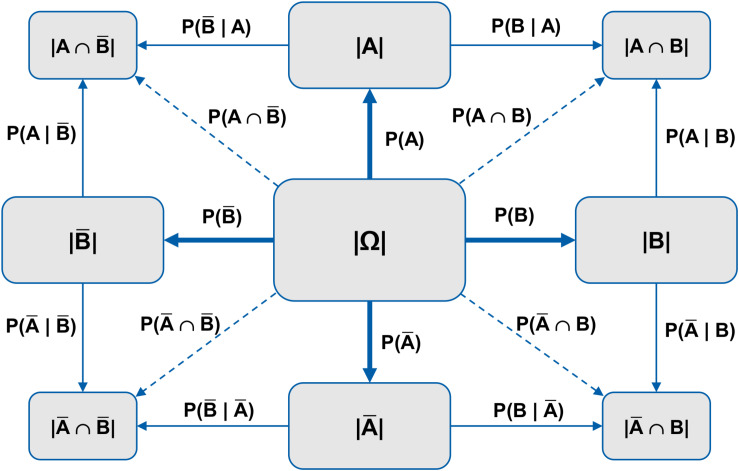
Schematic net diagram for two abstract events *A* and *B* and their counter-events $\bar{A}$ and $\bar{B}$, representing four marginal probabilities, four joint probabilities, and eight conditional probabilities.

**FIGURE 3:**
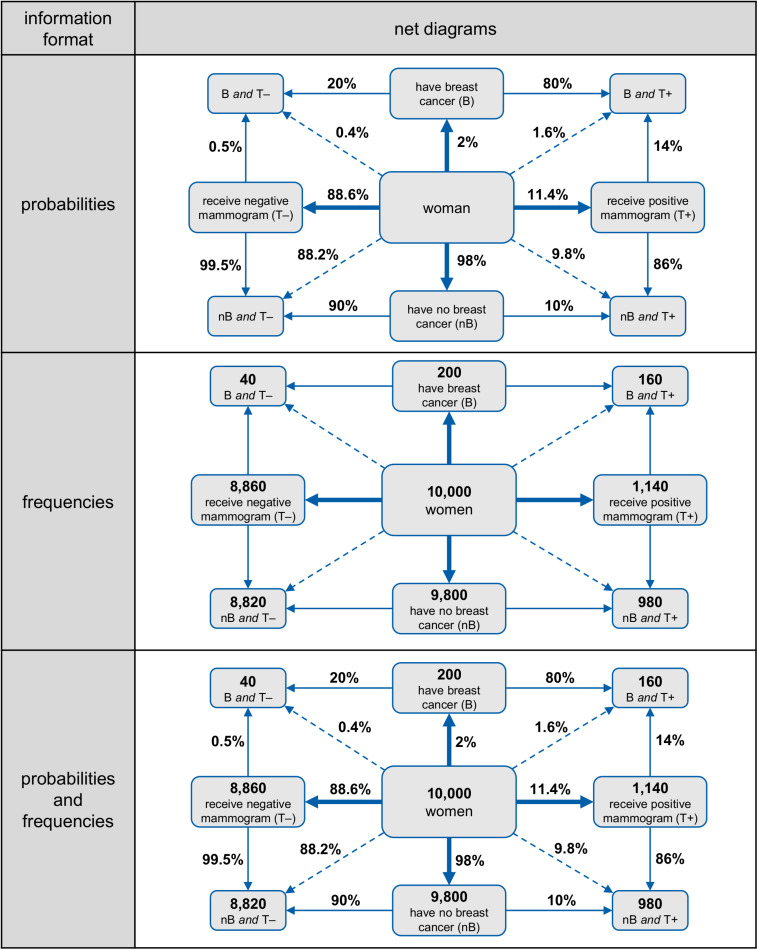
Net diagram with probabilities (top), frequencies (middle), or both information formats simultaneously for the mammography problem.

This article presents a novel visualization that enables the four marginal probabilities, all four joint probabilities, and all eight conditional probabilities to be taken in at a glance*: the frequency net.* Figure 2 shows a schematic net diagram for two abstract events *A* and *B*, and their respective counter-events $\bar{A}$ and $\bar{B}$. Moreover, in Figure 3, net diagrams (with probabilities, absolute frequencies, and both information formats) concerning the mammography problem are displayed. For a visualization coming close to our frequency net, yet without including joint probabilities (or corresponding branches), see Soto-Andrade (2019), and for a similar schematic visualization without joint probabilities or any numbers, see Wikipedia (without date)^1^.

It has to be noted that absolute frequencies and probabilities can be displayed simultaneously in net diagrams (see Figure 3, below). Therefore the frequency net, consisting of a node-branch structure, is an enhancement of a double-tree. As in the double-tree, all four marginal probabilities and all eight conditional probabilities can be depicted. In addition and in contrast to the double-tree, the net diagram has four branches for the joint probabilities. Furthermore, and also in contrast to the double-tree, no branches cross each other, and none of the nodes appears twice.

**FIGURE 4:**
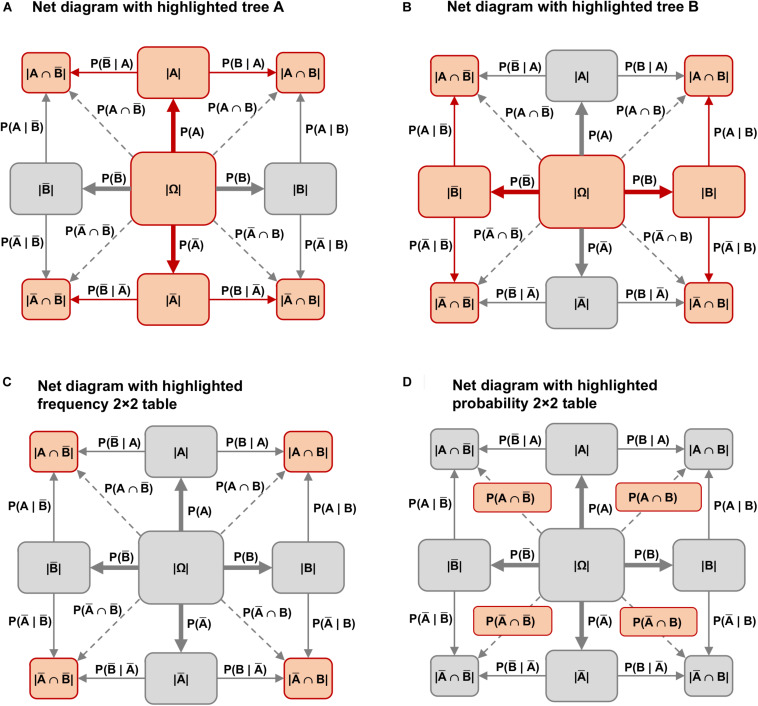
Both possible tree diagrams and the 2 × 2 table are included in the net diagram. **(A)** Net diagram with highlighted tree A; **(B)** Net diagram with highlighted tree B; **(C)** Net diagram with highlighted frequency 2 × 2 table; **(D)** Net diagram with highlighted probability 2 × 2 table.

The frequency net can also be seen as a hybrid version of a tree diagram combined with a 2 × 2 table: On the one hand, the frequency net consists of two tree diagrams that have been carefully placed one on top of the other (see Figures 4A,B; the two possible tree diagrams are also represented in a double-tree). On the other hand, the frequency 2 × 2 table is included in the four corner nodes (Figure 4C) of the net diagram, and the probability 2 × 2 table is included on the four branches for the corresponding joint probabilities (Figure 4D).

In the middle node of the net diagram (Figure 2), an (imaginary) sample size is displayed to which all further statistical information refers. First, the four marginal probabilities can be found from the middle node horizontally and vertically: P(*A*), P(*B*), P($\bar{A}$) and P($\bar{B}$). Second, the joint probabilities are plotted diagonally from the middle node to the corner nodes: P(*A*∩*B*), P($\bar{A}$∩*B*), P(*A*∩$\bar{B}$), P($\bar{A}$∩$\bar{B}$). Finally, the eight conditional probabilities are found at the borderlines of the net diagram: P(*A*|*B*), P($\bar{A}$|*B*), P(*A*|$\bar{B}$), P($\bar{A}$|$\bar{B}$), P(*B*|*A*), P($\bar{B}$|*A*), P(*B*|$\bar{A}$), and P($\bar{B}$|$\bar{A}$).

Note that in the net diagram, the following four probability rules apply, which are described separately in detail for probabilities and frequencies in Binder et al. (under review):

• *Line rule:* The sum of probabilities on opposing horizontal or vertical branches, both starting from the middle node is always 1.
• *Triangle rule (≙ multiplication rule in the tree diagram):* If you multiply the probabilities of the two “legs” in the eight elementary right-angled triangles, you get the probability displayed on the dashed hypotenuses.
• *V-rule (≙ addition rule in the tree diagram):* The sum of the probabilities of two adjoining diagonal (dashed) branches always equals the probability that is displayed on the enclosed branch [e.g., P(*A*∩*B*) + P(*A*∩$\bar{B}$) = P(A)].
• *X-rule:* The probabilities on all four diagonal branches added together result in 1.

Since we present in our results not only the performance of participants but also an analysis of their errors, we will consider in the next section prior research results concerning error analyses in Bayesian reasoning.

### Typical Errors in Bayesian Reasoning and Typical Errors With 2 × 2 Tables

#### Typical Errors in Bayesian Reasoning

From an educational point of view, it seems obvious to examine participants’ performance in relation to different information formats or visualizations. Equally interesting, however, is analyzing the reasons why participants were *not* able to solve a given task. In fact, many statistics educators, and also the psychologist McDowell and the statistician Jacobs, stress the importance of examining erroneous cognitive algorithms in Bayesian reasoning (McDowell and Jacobs, 2017). Weber et al. (2018), for example, found that many people who could not solve Bayesian reasoning tasks in the natural frequency format had first converted the statistical information back into probabilities and then subsequently failed in solving the task correctly. Lehner and Reiss (2018); Reani et al. (2018), and Bruckmaier et al. (2019) examined decision-making strategies (e.g., in contingency tables) with the help of eye-tracking analysis and found that eye-tracking is a useful method for investigating correct and incorrect solution algorithms, based on certain probability visualizations.

However, the demanding effort that an eye-tracking study involves is not always necessary. In many cases, the tasks can be constructed in such a way that the wrong solution itself already makes the faulty solution algorithm apparent. Along these lines, Gigerenzer and Hoffrage (1995) classified the wrong answers given by participants in “write-aloud protocols” and identified typical wrong answers in pure text versions of Bayesian tasks (see Table 2; compare also Eichler et al., under review; Steckelberg et al., 2004; Zhu and Gigerenzer, 2006; Días and Batanero, 2009; Eichler and Böcherer-Linder, 2018; Bruckmaier et al., 2019). Table 2 summarizes the few existing classifications of incorrect Bayesian reasoning strategies. While Gigerenzer and Hoffrage (1995) describe the typical erroneous strategies based on probabilities, Zhu and Gigerenzer (2006) and Eichler and Böcherer-Linder (2018) choose an explanatory approach based on frequencies. Bruckmaier et al. (2019), however, merge these two types of error presentation.

**TABLE 2:** Correct solution and typical incorrect Bayesian strategies with regard to the correct solution “F out of D” in a typical Bayesian reasoning task (according to Gigerenzer and Hoffrage, 1995; Steckelberg et al., 2004; Zhu and Gigerenzer, 2006; Días and Batanero, 2009; Eichler and Böcherer-Linder, 2018; Bruckmaier et al., 2019).

	Probabilities (with b, c, d, etc.)	Frequencies (with A, B, C, etc.)
Correct solution (Bayesian)	k = f/d = b⋅j/(b⋅j + m⋅c)	F out of D = F out of (F + G)
**Incorrect Algorithm (Non-Bayesian)**		
Joint occurrence (Gigerenzer and Hoffrage, 1995)	f = b⋅j = d⋅k	F out of A
Fisherian/Representative thinking/Transposed conditional (Gigerenzer and Hoffrage, 1995; Zhu and Gigerenzer, 2006; Días and Batanero, 2009)	j = f/b	F out of B
Base rate only/Conservatism (Gigerenzer and Hoffrage, 1995; Zhu and Gigerenzer, 2006)	b	B out of A
Evidence only (Zhu and Gigerenzer, 2006)	d = f + g = b⋅j + c⋅m	D out of A = (F + G) out of A
Likelihood substraction (Gigerenzer and Hoffrage, 1995)	j – m = f/b – g/c	(F out of B) – (G out of C)
Pre-Bayes (Steckelberg et al., 2004; Zhu and Gigerenzer, 2006)	Not applicable	B out of D = B out of (F + G)
Correct positive rate/false positive rate (Steckelberg et al., 2004)	j/m	Not applicable
		

It has to be noted that the research findings obtained thus far are also consistent with the *alignment hypothesis*: Some presentations of statistical information create “a better alignment between presented and requested relationships, and this should facilitate the comprehension of the requested ratio beyond the represented quantities” (Tubau et al., 2019, p. 1808; see also Johnson and Tubau, 2017). One common error in the text-only version of Bayesian reasoning problems is the *Fisherian*. In a frequency version, this error occurs because participants are mapping presented numbers onto the requested ratio without a proper comprehension of the relevant relationships.

**FIGURE 5:**
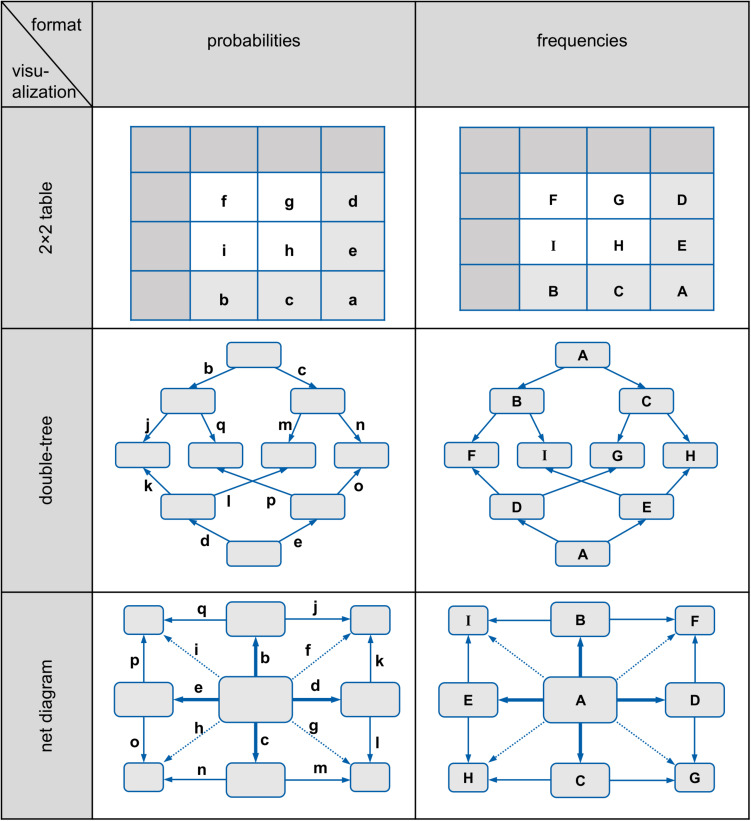
Schematic representation of 2 × 2 tables, double-trees, and net diagrams (left in probabilities, right in frequencies).

To this date, there has only been limited research on how error patterns shift when (1) the information format is changed, and (2) an additional visualization is shown. Such results are still lacking, especially with regard to non-Bayesian questions such as the one for joint probabilities. However, it has been understood since Gigerenzer and Hoffrage (1995) that in the pure text versions of Bayesian tasks, the errors *joint occurrence* and *Fisherian* are to be expected in both information formats. Furthermore, Bruckmaier et al. (2019) found evidence in an eye-tracking study that the *joint-occurrence* error appears more frequently in a probability 2 × 2 table than in a frequency 2 × 2 table. All errors of Table 2 are related to the notation that is shown in Figure 5 (uppercase letters stand for absolute frequencies while lowercase letters represent probabilities, see also Bruckmaier et al., 2019).

### Typical Errors With 2 × 2 Tables

Besides the above-mentioned studies on typical errors in Bayesian reasoning, there are several studies on typical errors and strategies regarding non-Bayesian judgments made with the use of a 2 × 2 table, for instance depending on different developmental stages in childhood (Batanero et al., 1994; Lehner and Reiss, 2018). Most of these studies focus on situations in which the proportion “F out of D” has to be compared with “I out of E” in 2 × 2 tables with frequencies (e.g., “Which one is larger?”). While there are several correct multiplicative strategies for solving this task (McKenzie, 1994), there are also various additive strategies that generally do not correspond to valid modeling of the situation and therefore can lead to misjudgments (Shaklee and Hall, 1983; Ufer et al., 2011; Lehner and Reiss, 2018). The present article, however, focuses on “simpler” inferences than the one just described. Instead of those complex comparisons of two different distributions, fewer mental steps are required for answering the questions in the present empirical study. The studies mentioned above are more about “read beyond the data,” whereas the present study is more about “read between the data” (Curcio, 1989). To the best of our knowledge, there are no comprehensive studies concerning typical difficulties in the simple act of choosing a number or a piece of information or even making simple inferences from a 2 × 2 table (for a study on the subject, albeit with only a few participants, see Bruckmaier et al., 2019). Furthermore, there are no studies on the efficiency of the frequency net thus far.

## Research Questions

The main goal of the present study is to examine empirically whether the net diagram can already be understood intuitively by participants without any prior explanation. We will explore the following research questions regarding the new visualization.

*Research question 1:* Depending on the information format (probabilities vs. frequencies), what effect do various visualizations (text only vs. 2 × 2 table vs. double-tree vs. net diagram) have on the ability of participants to solve a

a) conditional probability task?
b) joint probability task?

With respect to (a), we expect that all visualizations depicted with frequencies will have a positive effect on participants’ performance. Since 2 × 2 tables are taught in secondary school (in Germany) but double-trees and net diagrams are not, this study cannot deliver a fair direct comparison of these visualizations. Rather, this study is intended to test the hypothesis that the net diagram—although structurally completely unknown—is already as supportive to participant understanding as the other two visualizations.

Since no previous research results are available on question (b), it is rather explorative in nature. However, due to the frequent confusion of conditional probabilities with joint probabilities in typical Bayesian reasoning problems, we expect the opposite confusion to occur regarding the question for joint probabilities and assume that some participants will answer this question erroneously with a conditional probability.

Research question 2:

What is the effect of all three visualizations—again depending on information format—on specific errors that typically appear when asking for

a) conditional probability?
b) joint probability?

Do certain visualizations prevent or provoke specific errors? Since Bruckmaier et al. (2019) have already found, in an eye-tracking study with 24 participants, first indications that the 2 × 2 table with probabilities, for example, provokes the *joint-occurrence* error, we would like to examine this hypothesis in particular.

We also expect to find other erroneous strategies than those typical mistakes reported thus far because the presentation of a (fully completed) 2 × 2 table, a double-tree, or a net diagram show more statistical information than a tree diagram or purely textual Bayesian tasks, and therefore makes other typical error patterns possible.

## Empirical Study

### Design

In a paper-and-pencil questionnaire, participants were presented with two situations that are typical for Bayesian reasoning problems, the mammography problem and a short version of the economics problem (Ajzen, 1977; for problem formulations, see Table 5). The statistical information was either given in the structure of a typical Bayesian task (i.e., base rate, sensitivity, and false-alarm rate), or within a visualization (without any additional text provided with the statistical information). The presented diagrams were completely filled with numbers (either with frequencies or with probabilities). Therefore, in most cases, participants simply had to choose the correct number/pair of numbers, and no genuine inference was necessary (see Table 4).

The design of the study (see Table 3) includes two factors of interest (visualization and format of information) and one factor that was not of interest (context), resulting in a 4 × 2 × 2 design:

**TABLE 3:** Design of the 16 tested problem versions.

		Context	
		Mammography problem	Economics problem
**Information format**	**Probabilities**	• Bayesian text • 2 × 2 table • double-tree • net diagram	• Bayesian text • 2 × 2 table • double-tree • net diagram

	**Frequencies**	• Bayesian text • 2 × 2 table • double-tree • net diagram	• Bayesian text • 2 × 2 table • double-tree • net diagram

*Two questions were asked for each of the 16 versions: The first question addressed a conditional probability and the second question addressed a joint probability.*

• *Factor 1: Visualization:* Bayesian text vs. 2 × 2 table vs. double-tree vs. net diagram.
• *Factor 2: Format of information:* probabilities vs. frequencies.
• *Factor 3: Context:* mammography problem vs. economics problem (not a factor of interest).

Each participant received one of the two problem contexts with probabilities and the other with frequencies. In that way, the order of context and information format was varied systematically. Furthermore, if, for instance, in one of the two problems a 2 × 2 table was displayed, in the other problem either no visualization, a double-tree, or a net diagram was presented. Note that in the versions with visualizations, the text with the statistical information was *not* presented additionally, so that participants had to use the visualization. A former study showed no effect on participants’ performance whether one provides the text with an additional visualization or not (Binder et al., 2018). Because with the text version it is only possible to formulate text with either conditional probabilities *or* joint probabilities (compare standard menu vs. short menu in Gigerenzer and Hoffrage, 1995), we decided to provide only “Bayesian text versions” (i.e., no text with joint probabilities), which is more in line with previous research. The amount of information given is therefore different in each version: Each of the Bayesian text versions consists of three pieces of information, but note that the three pieces of information in the natural frequency version are composed of five absolute frequencies. The net diagram used in our study displayed all 16 probabilities in the probability version (see Figure 3, above), or all 9 frequencies in the frequency version (see Figure 3, middle). In the frequency 2 × 2 table, frequency double-tree, and frequency net *all nine* absolute frequencies are displayed. Whereas the probability 2 × 2 table shows only joint probabilities (in addition to marginal probabilities), the double-tree displays only conditional probabilities (in addition to marginal probabilities). However, with the net diagram implemented in our study, one can see *all* 16 probabilities at a glance.

In Table 3 the design of the study is illustrated, resulting in 16 implemented versions, and in Table 5 the corresponding problem formulations are denoted. In each of the 16 versions, two different questions were asked: The first question addressed a conditional probability and the second question addressed a joint probability.

Note that in contrast to many other studies, our tasks do not require a genuine inference and thus fewer mental steps are required (with the exception of the Bayesian text versions; compare Ayal and Bayth Marom, 2014). Since the visualizations already provide a good deal of statistical information, in many cases only the matching number(s) has (have) to be chosen from the visualization (i.e., in the one case the requested probability and in the other case the two absolute frequencies that form the corresponding natural frequency). In the following we will only speak of something as a genuine inference if it was not enough simply to select one or two numbers but instead was necessary to combine further numbers, for example, with addition, subtraction, multiplication, or division being necessary to solve the problem.

Table 4 displays the requested cognitive strategies for answering the implemented tasks. Since Ayal and Bayth Marom (2014) have shown that participants perform poorly on complicated tasks that require more mental steps, we distinguish three different levels of complexity in Table 4. Whereas in the Bayesian text versions a genuine inference is always necessary, in most other versions it is sufficient to identify and choose the correct number (in probability versions) or the correct pair of numbers (in frequency versions), which is much easier than making a genuine inference because it requires fewer mental steps. However, according to Cognitive Load Theory (Sweller, 2003), it is probably not so easy to find the right number among many numbers.

**TABLE 4:** Mental steps that are necessary for answering each question.

		Required for answering	
	Visualization	Question for a conditional probability/frequency	Question for a joint probability/frequency
**Probabilities**	**Bayesian text**	Genuine inference necessary	Genuine inference necessary
	**2 × 2 table**	Genuine inference necessary	Choose a number (probability)
	**double-tree**	Choose a number (probability)	Genuine inference necessary
	**net diagram**	Choose a number (probability)	Choose a number (probability)

**Frequencies**	**Bayesian text**	Genuine inference necessary	Genuine inference necessary
	**2 × 2 table**	Choose a pair of numbers (frequencies)	Choose a pair of numbers (frequencies)
	**double-tree**	Choose a pair of numbers (frequencies)	Choose a pair of numbers (frequencies)
	**net diagram**	Choose a pair of numbers (frequencies)	Choose a pair of numbers (frequencies)

It has to be noted that whereas all university students are already familiar with 2 × 2 tables from secondary school, most have never seen a double-tree or a net diagram before. It should also be noted that a question asking for natural frequencies is unusual in German secondary education.

Please note that the main focus of the present empirical study is the question of conditional probabilities. In the current study, the order of questions for conditional probabilities and joint probabilities is *not* varied systematically (in that case, twice as many participants would have been required.). This could influence the responses of the participants who have already answered a question about conditional probabilities, for example.

There were no time constraints for completing the questionnaire (participants required about 20 min for both tasks). Participants were examined in small groups of about 10–20 persons. Pocket calculators were distributed, which could be used at any time during the study.

### Instrument

Each participant was presented two successive tasks that varied in terms of (1) visualization (Bayesian text vs. 2 × 2 table vs. double-tree vs. net diagram), (2) information format (probabilities vs. frequencies), and (3) problem context (mammography vs. economics problem). All versions began with a cover story (see also Table 5); after that, one of the four different kinds of visualizations (including no visualization) was given (see Figure 1 above and below for the 2 × 2 tables and the double-trees, and see Figure 3 above and in the middle row for the net diagrams for the mammography context). Finally, two questions were provided in the same format as the information in the text: One question for a (Bayesian) conditional probability/frequency and one question for a joint probability/frequency (see Table 5).

**TABLE 5:** Problem formulations.

	Mammography problem		Economics problem	
	Probability version	Natural frequency version	Probability version	Natural frequency version
**Cover story**	Imagine you are a reporter for a women’s magazine and you want to write an article about breast cancer. As a part of your research, you focus on mammography as an indicator of breast cancer. You are especially interested in the question of what it means when a woman has a positive result (which indicates breast cancer) in such a medical test. A physician explains the situation with the following information:		Imagine you are interested in the question, of whether career-oriented students are more likely to attend an economics course. Therefore the school psychological service evaluates the correlations between personality characteristics and choice of courses for you. The following information is available:	

**Visualization**	• Text only (no visualization): The probability of breast cancer is 2% for a woman who participates in routine screening. If a woman who participates in routine screening has breast cancer, the probability is 80% that she will have a positive test result. If a woman who participates in routine screening does not have breast cancer, the probability is 10% that she will have a positive test result.	• Text only (no visualization): 200 out of 10,000 women who participate in routine screening have breast cancer. Out of 200 women who participate in routine screening and have breast cancer, 160 will have a positive result. Out of 9,800 women who participate in routine screening and have no breast cancer, 980 will also have a positive result.	• Text only (no visualization): The probability that a student attends the economics course is 32%. If a student attends the economics course, the probability that he is career-oriented is 64%. If a student does not attend the economics course, the probability that he is still career-oriented is 60%.	• Text only (no visualization): 320 out of 1,000 students attend the economics course. Out of 320 students who attend the economics course, 205 are career-oriented. Out of 680 students who not attend the economics course, 408 are still career-oriented.
	• 2 × 2 table (prob.), or • double-tree (prob.), or • net diagram (prob.)	• 2 × 2 table (nat. freq.), or • double-tree (nat. freq.), or • net diagram (nat. freq.)	• 2 × 2 table (prob.), or • double-tree (prob.), or • net diagram (prob.)	• 2 × 2 table (nat. freq.), or • double-tree (nat. freq.), or • net diagram (nat. freq.)

**Question 1 – cond. prob.**	What is the probability that a woman who participates in routine screening and receives a positive test result has breast cancer?	How many of the women who participate in routine screening and receive a positive test result have breast cancer?	What is the probability that a student attends the economics course if he is career-oriented?	How many of the students who are career-oriented attend the economics course?
	Answer: ____ out of ____	Answer: _______	Answer: ___ out of ____	Answer: _______

**Question 2 – joint prob.**	What is the probability that a woman who participates in routine screening receives a negative test result *and* has breast cancer?	How many of the women who participate in routine screening receive a negative test result *and* have breast cancer?	What is the probability that a student attends the economics course *and* is not career-oriented?	How many of the students are not career-oriented *and* attend the economics course?
	Answer: _______	Answer: ____ out of ____	Answer: _______	Answer: ____ out of ____

### Participants

Participants were *N* = 249 German university students in the fields of Pharmacy (*N* = 117), Human Movement Sciences (*N* = 33), student teacher for primary school (*N* = 90), and student teacher for secondary school (*N* = 9). 184 students were female, 65 male, and the mean age value was 20.6 (*SD* = 2.2). From their secondary school education, all students were familiar with 2 × 2 tables containing probabilities, 2 × 2 tables containing frequencies, and tree diagrams containing probabilities, yet not with tree diagrams containing absolute frequencies, double-trees, or net diagrams.

The study was carried out in accordance with the University Research Ethics Standards. Students were informed that their participation was voluntary (two students refrained from participating) and anonymity was guaranteed.

### Coding

#### Conditional Inferences

The correct solution for the mammography problem in the frequency version is 160 out of 1,140 and for the economics problem 205 out of 613. The answer was coded as correct if both correct absolute numbers were provided. In the probability versions of the tasks, the answer was classified as correct if the exact probability was provided (14.03% in the mammography problem and 33.4% in the economics problem). In addition, the answers were also coded as correct if the solution was rounded up or down to the next full percentage point (e.g., in the economics problem the correct solution is 33.4%, and therefore answers between 33 and 34% were classified as a correct solution; see also Gigerenzer and Hoffrage, 1995). To be conservative, we also coded the answer as correct if the solution algorithm was correctly specified but no final result was calculated.

#### Joint Inferences

The correct solution for the mammography problem in the frequency version is 40 out of 10,000 and for the economics problem 115 out of 1,000. Again, the answer was only coded as correct if both correct absolute numbers were provided in the frequency version. In the probability versions of the tasks, the correct answer of the mammography problem is 0.4%, and every answer between 0.4% and 0.5% (but exclusive of 0.5% because 0.5% was one of the expected wrong solutions) was coded as correct. In the economics problem, the correct solution was 11.5%, and every answer between 11% and 12% was coded as correct. We have also classified the answer 0.1 as correct for two participants because it was clearly recognizable that the solution algorithm was correct and the result was only incorrectly rounded. In these two cases it was a Bayesian text version with probabilities and a version with a probability net. The classification of these two answers as correct was therefore conservative against our research question.

## Results

### Participants’ Performance With Respect to Conditional Inferences

**FIGURE 6:**
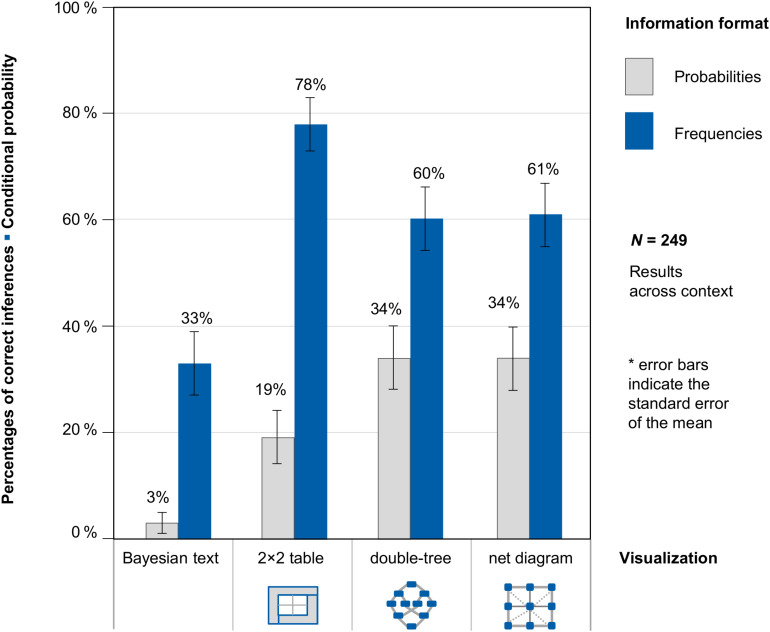
Percentages of correct inferences in the question for a conditional probability, separated for information format and visualization type (across both contexts).

Figure 6 shows participants’ performance on the question for conditional probabilities across contexts (because context was no factor of interest in our study). Supplementary Figure S1, however, shows participants’ performance on the question for conditional probabilities, separately for the two different contexts (mammography problem vs. economics problem).

With regard to the question for conditional probability, two relevant results can be observed. First, students performed better when statistical information was presented in frequencies (58% correct inferences across context and visualization) than in probabilities (23% correct inferences across context and visualization). This finding holds true for both contexts and for all three visualizations. Second, the presentation of a visualization leads to higher performance rates (48% correct inferences) compared to a Bayesian text problem (19% correct inferences; again holding true across all versions and conditions).

As expected, the highest performance was achieved in problems using the only visualization participants knew from secondary school lessons: the 2 × 2 table with frequencies (78% correct inferences). However, participants also performed very well with the frequency double-tree and the frequency net (60 and 61% correct solutions), which students had not encountered in their secondary education. The more mental steps required for answering the question correctly, the lower the performance rate.

In order to statistically compare the effects of information format and types of visualization, we estimated a generalized linear mixed model with a logit link function to predict performance regarding the question for a conditional probability. In this model, we specified the probability version without any visualization (Bayesian text in probabilities) as the reference category and included the possible explanatory factors “frequencies,” “2 × 2 table,” “double-tree,” and “net diagram” via dummy coding.

The (unstandardized) regression coefficient for frequencies was significant (*b*_1_ = 1.88, *SE* = 0.27, *z* = 6,97, *p* < 0.001), and presenting a corresponding 2 × 2 table (*b*_2_ = 1.81, *SE* = 0.36, *z* = 4.99, *p* < 0.001), double-tree (*b*_3_ = 1.76, *SE* = 0.37, *z* = 4.78, *p* < 0.001), or net diagram (*b*_4_ = 1.77, *SE* = 0.36, *z* = 4.91, *p* < 0.001) also led to a significant regression coefficient (*b*_0_ = −2.83, *SE* = 0.38, *z* = −7.39, *p* < 0.001). Thus with regard to conditional inferences, frequencies and all visualizations were helpful for solving the task.

Furthermore, the actual level of education (“Semesterzahl”), grade point average (German “Abiturnote” from high school), and field of study were collected from all participants. These variables (and also the context of the task: mammography problem vs. economics problem) were then implemented as potential predictors in the generalized linear mixed model. It turned out that the context, the grade point average, and studying to be a secondary school teacher significantly predicted the probability of solving a conditional inference correctly. However, implementing these factors in the generalized linear mixed models did not change the results substantially. Furthermore, there were no significant order effects. However, there was a slight tendency for the second task (joint probability/frequency) to be correctly completed less frequently than the first task (conditional probability/frequency), as has been shown in earlier studies (Binder et al., 2018).

Although this article does not do any in-depth comparison of the different visualizations (because one visualization was known for the participants, the others two were not), it can be noted that performance on tasks using double-tree and net diagram are remarkably high given the fact that neither of these two visualizations has been explained in advance.

### Participants’ Performance With Respect to Joint Inferences

**FIGURE 7:**
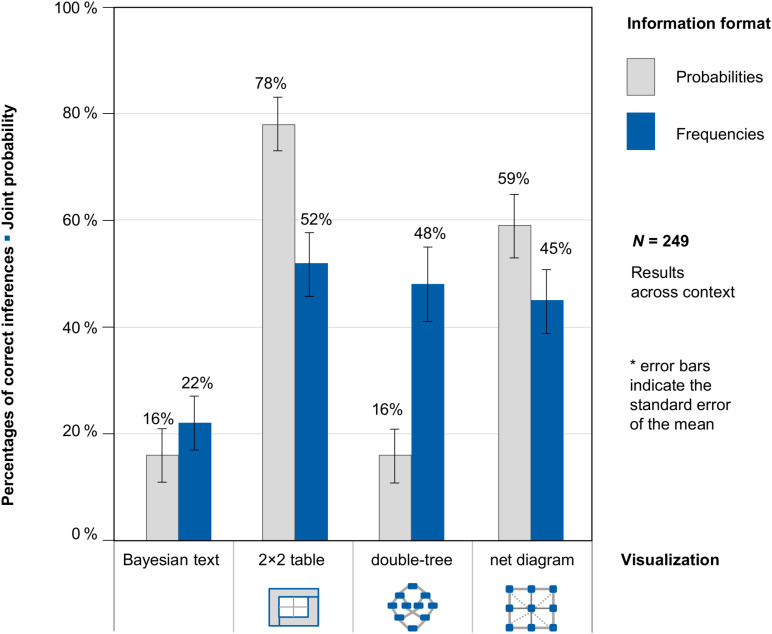
Percentages of correct inferences in the question for a joint probability, separated for information format and visualization type (across both contexts).

Figure 7 shows participants’ performance with respect to joint probabilities and frequencies across context (because context was no factor of interest in our study). Supplementary Figure S2, however, shows participants’ performance with respect to joint inferences, separately for the two different contexts (mammography problem vs. economics problem).

The study shows three interesting results: (1) If the frequency versions only are considered, each visualization of the situation was similarly helpful for the participants—no matter which of the visualizations (2 × 2 table, double-tree, or net diagram) was used. (2) In contrast to conditional inferences in typical Bayesian reasoning problems, the question of joint probabilities does not reveal a format effect (probabilities vs. frequencies). Tasks with frequencies were not processed better than tasks with probabilities. Possible reasons for this differential format effect are outlined in the discussion. (3) The highest performance was reached with probability 2 × 2 tables (which is in line with Bruckmaier et al., 2019; Binder et al., under review) and probability nets. Note that in these versions, the number of mental steps required is also the fewest. In both cases only one number has to be read from the diagram, while in the double-tree a genuine inference is required (compare also Table 4).

Note again that the main focus of the present empirical study was *conditional inferences*. The order of questions for conditional probabilities and joint probabilities was *not* varied systematically. This could have influenced the responses of participants who had already answered a question about conditional probabilities before the question on joint probabilities.

Again, in order to statistically compare the effects of information format and type of visualization, we estimated a generalized linear mixed model with a logit link function to predict performance in a joint probability question.

This time, the (unstandardized) regression coefficient for frequencies was not significant (*b*_1_ = −0.05, *SE* = 0.19, *z* = −0.26, *p* = 0.80), but presenting a 2 × 2 table (*b*_2_ = 2.04, *SE* = 0.31, *z* = 6.53, *p* < 0.001), double-tree (*b*_3_ = 0.69, *SE* = 0.30, *z* = 2.28, *p* = 0.02), or net diagram (*b*_4_ = 1.53, *SE* = 0.30, *z* = 5.09, *p* < 0.001) led to a significant regression coefficient (*b*_0_ = −1.41, *SE* = 0.26, *z* = −5.46, *p* < 0.001). Thus regarding joint inferences, 2 × 2 tables and net diagrams were most helpful. Also, double-trees led to a significantly higher performance rate compared to a Bayesian textual version of the task. However, there is no frequency effect in joint inferences.

Again, the level of education, grade point average, and field of study of the participant, as well as the context and the order of the task, were implemented as potential predictors in the generalized linear mixed model. We found that only the grade point average significantly predicted the probability of a joint inference being correct. However, implementing these factors in the generalized linear mixed models did not change the results. Furthermore, there were no significant effects of order, context, level of education, or field of study.

### Typical Errors and Error Shifts Regarding Conditional Inferences

**FIGURE 8:**
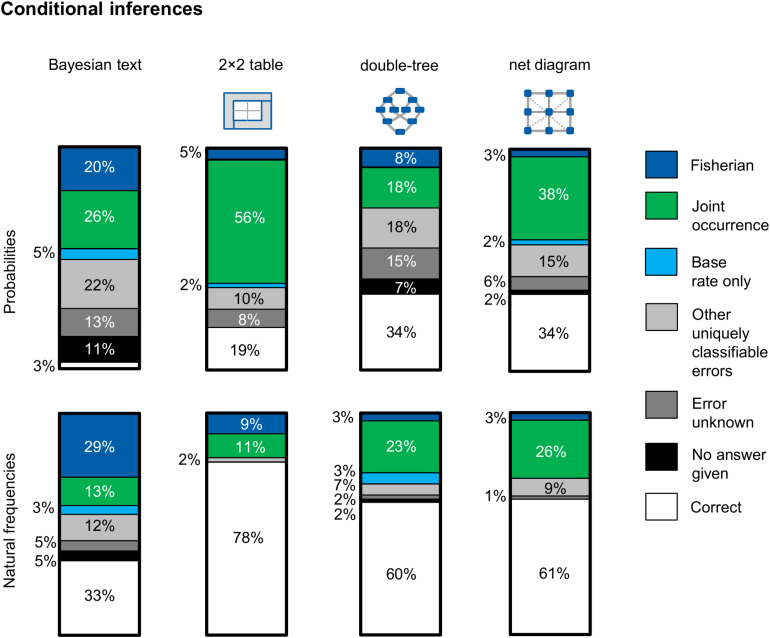
Typical errors on the question for a conditional probability, separated for information format and visualization type (across both contexts). In particular, the two errors *Fisherian* and *joint-occurrence* could be observed.

Figure 8 shows—separated by version—the respective errors that occurred regarding conditional inferences. Note that only errors that occurred in at least 5% of the cases in one of the examined versions are mapped in Figure 8. This means in concrete terms that if one error occurred in one version (e.g., in the Bayesian text version with probabilities) in 5% of the cases or more (e.g., the error “base rate only”), this error is also displayed for all other versions (even if this error only occurs in 2% of the cases in the probability net). Other errors that can be clearly classified but which were committed by only one or two participants per version are thus assigned to the category, “Other uniquely classifiable errors.”

Essentially, participants made two main mistakes regarding conditional inferences: (1) *joint occurrence*, which is the confusion of the conditional information P(A|B) with the joint information P(A∩B) [e.g., indicating the proportion of women with a positive mammogram *and* breast cancer P(T+∩B) instead of the correct conditional information P(B|T+)], and (2) *Fisherian*, which means that participants confused P(A|B) with P(B|A) [e.g., indicating the sensitivity of the mammography P(T +|B) as the correct solution instead of the positive predictive value P(B|T+)]. Furthermore, in some cases the *base rate only* error occurs, which means providing only the base rate of, for example, breast cancer P(B) as an answer. This error most often appeared in the Bayesian text version in probabilities. It is noticeable that most of the wrong solution strategies could be clearly classified. The errors *evidence only* (see, e.g., Zhu and Gigerenzer, 2006) and *Pre-Bayes* (see, e.g., Steckelberg et al., 2004; Zhu and Gigerenzer, 2006) only occurred very rarely. In contrast to Gigerenzer and Hoffrage (1995), who sometimes observed the error *likelihood-substraction* (especially in probability versions), that error did not occur in our study. In the Bayesian text version with probabilities, there was (as one would expect) the highest proportion of participants who could not give a solution (11%).

The analysis of the error pattern in Figure 8 shows three main results: First, an interesting result (according to our hypothesis) is obtained by comparing the probability 2 × 2 table with the frequency 2 × 2 table. Bruckmaier et al. (2019) have already provided evidence that the probability 2 × 2 table provokes the *joint-occurrence error*, which we replicated (56% of the participants). The error rate, on the other hand, drops considerably if the information is presented in frequencies (only 11% of the participants made this error when the information was presented in this way). It seems as if it is not at all clear to many participants that the joint probability in the probability 2 × 2 table must be associated with another number. With frequencies, however, this necessity does seem to be clear to participants.

Second, if one compares all probability visualizations, it becomes clear that the *joint-occurrence error* appears primarily in versions in which the joint probability is directly represented—most frequently in the 2 × 2 table and second most frequently in the net diagram—because the correct solution is also shown there. As expected, the joint-occurrence error appears most rarely in the version with the probability double-tree. The reason why that error is comparatively rare in this version is that the joint probability first has to be calculated using the multiplication rule in the double-tree.

Third, if one compares all frequency visualizations, a shift of Fisherian and joint-occurrence errors can be observed. While Fisherian and joint-occurrence errors appear about as frequently in the 2 × 2 table, Fisherian errors in the frequency double-tree and in the frequency net hardly occur at all. A confusion of the “reading direction” is therefore less frequent in the frequency double-tree and the frequency net. In these two visualizations, however, joint occurrence appears more frequently. It seems less clear to participants that the total number should not be chosen as the reference set. It should be a focus of future research to investigate the extent to which the error patterns change after a training with the different visualizations.

### Typical Errors and Error Shifts Regarding Joint Inferences

**FIGURE 9:**
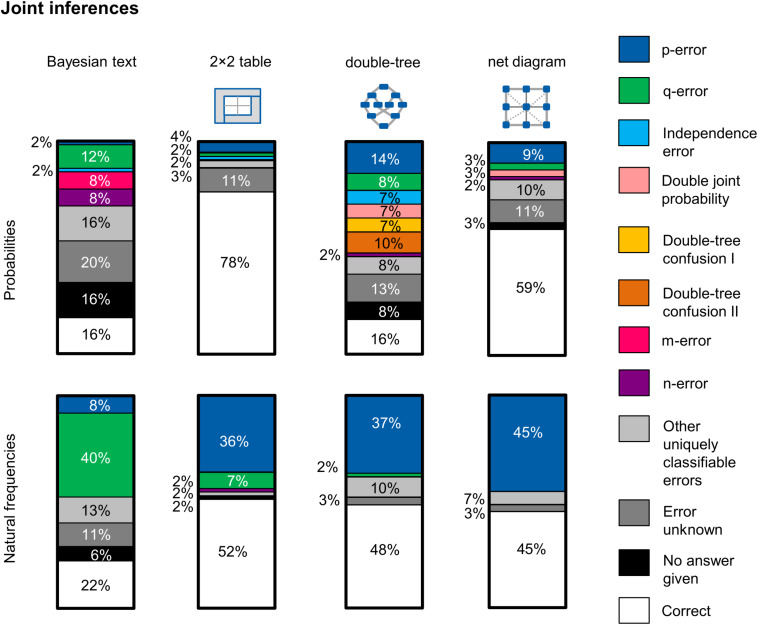
Typical errors on the question for a joint probability, separated for information format and visualization (across both contexts). In the versions with frequencies, two main errors can be observed: the confusion of the joint probability either with the conditional probability *p* or the conditional probability *q*. In the versions with probabilities, on the other hand, more diverse error patterns appear: Specific errors are provoked by the pure text version with probabilities and the probability double-trees.

While typical error patterns for conditional inferences are already recognized because of earlier research, we now systematically consider the error patterns regarding joint inferences. Figure 9 shows—separated by version—the respective errors. When naming these errors, we refer to the expressions from Figure 5 [e.g., the expression “p-error” means that the participant erroneously answered the question with P(B|T–) instead of P(B∩T–)]. Again, only errors that occurred in at least 5% of the cases in one of the examined versions are mapped in Figure 9. This means in concrete terms that if one error occurs in one version (e.g., in the Bayesian text version with probabilities) in 5% of the cases or more (e.g., the “m-error”), this error is also displayed for all other versions (even if this error only occurs in 3% of the cases in the probability net). Errors that can be clearly classified but which were committed by only one or two participants are again grouped in the category “Other uniquely classifiable errors.”

Figure 9 shows three results: First, if we look at the frequency versions, we can see at a glance that the error patterns are less diverse than they are in the probability versions. In the frequency versions two errors occur often: the confusion of the joint probability [e.g., P(T–∩B)] with one of the two corresponding conditional probabilities [i.e., P(T–|B) = : *q-error* and P(B|T–) = : *p-error*; these errors are structurally equivalent to the joint-occurrence error]. Actually, it should be assumed that the p-error occurs as frequently as the q-error because there is no reason why P(A∩B) should be confused with P(A|B) rather than P(B|A). However, it is understandable that the q-error occurs more frequently in the Bayesian text version, because this error is algorithmically easier to calculate in this version. But in the three versions with a visualization, this argument is no longer valid. Both conditional probabilities, p-error and q-error, can now be read from the diagrams with equal ease. Here we have defined a clear reading direction for the visualizations by the nature of our question. We asked for the probability of “negative mammogram *and* breast cancer” and not for the probability “breast cancer *and* negative mammogram” (which is of course mathematically equivalent). The participants now seemed to examine the three visualizations along the lines of the question, which more often provokes the p-error. It would be interesting in a new study to vary the order of events in the question and, for example, to examine in an eye-tracking study whether our hypothesis is correct that the error patterns p-error and q-error are provoked by the order of events.

Second, it is noticeable that in the probability versions various error patterns appear—for example in the Bayesian text version with probabilities. In addition to a few errors that we could not classify, there are many error patterns that can be clearly classified but which occur only very rarely. However, two confusions occurred more frequently (both in the responses of 5 out of 61 persons): the confusion of the required joint probability with the conditional probabilities *m-error* [i.e., P(T+|nB)] and *n-error* [i.e., P(T–|nB)]. In these cases, participants obviously misread the negations in the question.

Third, specific errors can also be observed in the probability double-tree. This was to be expected, since in the probability versions more mental steps are necessary for solving the task with the double-tree, while the required joint probability can be read directly from the probability 2 × 2 table or from the probability net. The two double-tree confusions are particularly interesting here. We provoked these two confusions inadvertently by writing the two conditional probabilities *l* and *p* on the branches in such a way that it was difficult to see which of the two pieces of information belonged to which branch (as in Figure 1). We assume that both university and school students also often follow that path when drawing a double-tree and that these mistakes are therefore ecologically valid. However, we could have prevented this error by designing the double-tree as shown in Figure 5, so that confusion between the two branches *l* and *p* would be less likely. *Double-tree confusion I* consists of the fact that many of the participants confused the joint probability with the conditional probability *p* (like many other participants) and additionally confused the branches *p* and *l*. If we had designed the double-tree in such a way that the conditional probabilities *p* and *l* could be better distinguished, these participants would presumably have committed the p-error. *Double-tree confusion II*, on the other hand, would have led to the correct answer, because here the participants correctly calculated the joint probability using the multiplication rule. However, they confused the branches and thus the probabilities *p* and *l* and came to a wrong result. If we had made the lower branches in the double-tree clearer, these participants would probably have calculated the joint probability correctly.

In addition to these three main findings, the occurrence of the *independence error* is also interesting—here the participants calculated the joint probabilities by multiplying the associated marginal probabilities. The students probably remembered the formula P(A∩B) = P(A) ⋅ P(B) and did not consider that this formula only applies if the events are independent. Furthermore, some participants committed the *double joint probability error*, which means that they tried to calculate the joint probability from both above in the double-tree and below with the multiplication rule. Each of these calculations would lead to the right solution on its own. However, the participants then added these two results and came to a solution of exactly twice the probability they were looking for.

## Discussion

In this article the frequency net is presented as a new tool for simultaneously visualizing probabilities *and* frequencies, a capability that is not possible with the use of existing visualizations such as the 2 × 2 table, the tree diagram, and the double-tree. Whereas 2 × 2 tables only display joint probabilities but no conditional probabilities, tree diagrams and double-trees only display conditional probabilities but no joint probabilities. Before the frequency net, no visualization had the capacity to represent all 16 probabilities that can occur in a situation with two dichotomous characteristics (i.e., four marginal probabilities, four joint probabilities, and eight conditional probabilities) and all frequencies simultaneously. The fact that the frequency net can enable visualization of (1) probabilities *and* frequencies and (2) joint probabilities *and* conditional probabilities is a didactic advantage because performing demanding additional calculations based on a net diagram is no longer necessary.

In an empirical study conducted with university students, the net diagram was already as intuitively understandable (to a comparable degree) as the 2 × 2 table and the double-tree, even without prior explanations. In a similar way, Binder et al. (under review) could show that secondary school students (grade 10) were also able to use this tool intuitively without prior instruction, and that the net diagram even supported the students in this study in solving probability problems better than a tree diagram or a double-tree did.

An analysis of typical error patterns shows—regarding conditional inferences—a remarkable error shift from probability 2 × 2 tables to frequency 2 × 2 tables. Whereas many participants committed the *joint-occurrence error* with probability 2 × 2 tables, this error disappeared almost completely with the frequency 2 × 2 table. The analysis of errors regarding joint inferences—on which there have been only a few previous studies—reveals that the formulation of the question [P(A∩B) vs. P(B∩A)] seems to provoke either a q-error or a p-error (see also section Future Research). Furthermore, many different error patterns occurred in the Bayesian text version with probabilities. Especially interesting, however, were the errors specific to the double-tree, some of which were provoked by our having written the labels 86 and 0.5% in unfavorable positions on the crossing branches (so that these numbers could not unambiguously be assigned to the appropriate branches). It is very likely that these mistakes would also occur if participants were asked to create their own double-tree (because these positions in the double-tree seem like a good place to write these two conditional probabilities). Also interesting is the occurrence of the independence error and the double-joint probability error, which occur predominantly in the probability double-tree.

### Limitations

The present article and Binder et al. (under review) can of course only provide first indications of the efficacy of the net diagram in teaching probabilities—even though these first indications are very promising. In the teaching context, for example, learners have to be instructed that branches in the net can now also display joint probabilities (whereas the widely used tree diagrams only carry marginal probabilities and conditional probabilities).

Furthermore, it could also be argued that the presentation of the information in a frequency net does not make the sequential character of the situation as transparent as it is in a tree diagram. However, the error analysis does not indicate that the reading direction (*Fisherian error*) becomes confused more frequently with the net diagram than it does with the double-tree or even the 2 × 2 table (a bit more rare).

The main limitation of the net diagram is that it cannot be extended as flexibly as the tree diagram. Tree diagrams can be adapted to 2-test cases, 3-test cases, etc. (Hoffrage et al., 2015b; Binder et al., 2018). However, it should be noted that even the double-tree cannot be expanded flexibly to 2-test cases, 3-test cases, and other complex Bayesian reasoning tasks. In any case, these kinds of tasks are rarely the focus of teaching stochastics at the secondary-school level.

### Future Research

The missing format effect regarding joint inferences is at the same time interesting and unusual. One reason for its being missing, however, could be the formulation of the question and especially the required answer structure (“____ out of ____”) in the frequency format, which is unusual to students. In the teaching of stochastics at secondary-school level, two different types of questions are used: (1) Questions about a probability or a proportion, which students are expected to answer in percentages, fractions, or decimal fractions, or (2) in lower-level classes, questions like “How many are X and Y?” which students are expected to answer with an absolute frequency (e.g., “200”) but not with a pair of absolute frequencies, namely natural frequencies (e.g., “200 out of 10,000”). Therefore, it would be quite possible that the participants were confused by the unusual answer format (“____ out of _____”). Hence, for future research it would be interesting to examine whether there is actually a format effect if, for example, one asks for a probability or a proportion in the frequency version of the task.

Furthermore, in future research a systematic variation of the order of the questions (conditional probabilities vs. joint probabilities) should be implemented in order to identify any possible sequence effect. In the present study, the question for a conditional probability was always asked as the first question and the question for a joint probability as the second question. This could have influenced participants’ performance and also the errors that occurred in second-question responses, due to, for example, an Einstellung effect or a mental set effect (Luchins, 1942).

Future research should focus more on error analysis than just measuring the performance of participants in Bayesian reasoning. Furthermore, not just the typical Bayesian tasks should be examined but also other probability tasks that are focused on stochastic teaching in schools (see also Böcherer-Linder and Eichler, 2017; Bruckmaier et al., 2019). Moreover, in future research on the net diagram, it would be desirable to include additional control variables because various other factors are known to have an impact on performance in Bayesian reasoning tasks. For example, individual differences of participants, particularly cognitive abilities such as numeracy, graphicacy, and spatial abilities, have an impact on performance rates in Bayesian reasoning problems (e.g., Chapman and Liu, 2009; Micallef et al., 2012; Johnson and Tubau, 2013; Ayal and Bayth Marom, 2014). In addition, the length of the text (Johnson and Tubau, 2013) and the specific numerical values for population size, base rate, sensitivity, and false-alarm rate can influence accuracies (Schapira et al., 2001).

Since one advantage of the net diagram is that it can display both probabilities and frequencies, it would be interesting to implement in further studies a net diagram that displays both types of representation simultaneously (see Figure 3, below). It would also be important to examine net diagrams that only show the statistical information, which is needed for solving the task at hand (because that is the way it would be done at school).

In further investigations, training studies might be implemented, which are fairer in terms of existing prior knowledge of certain visualizations from school. With training studies is it possible to examine whether students are able to create frequency nets on their own by first explaining the structure of the frequency net to students and then have them drawing their own frequency nets for subsequent tasks.

## Data Availability Statement

The datasets generated for this study can be found in the repository of the University of Regensburg, https://epub.uni-regensburg.de/43053/.

## Ethics Statement

Ethical review and approval was not required for the study on human participants in accordance with the local legislation and institutional requirements. The participants provided their written informed consent to participate in this study.

## Author Contributions

All authors listed have made a substantial, direct and intellectual contribution to the work, and approved it for publication.

## Conflict of Interest

The authors declare that the research was conducted in the absence of any commercial or financial relationships that could be construed as a potential conflict of interest.

## Footnotes

1 https://commons.wikimedia.org/wiki/File:Bayes%27_Theorem_2D.svg

## References

[B1] Ajzen I. (1977). Intuitive theories of events and the effects of base-rate information on prediction. *J. Pers. Soc. Psychol.* 35 303–314. 10.1037/00223514.35.5.303

[B2] Ayal S. Bayth Marom R. (2014). The effects of mental steps and compatibility on Bayesian reasoning. *Judgment Decision Making* 9 226–242.

[B3] Baratgin J. (2015). Rationality, the Bayesian standpoint, and the Monty-Hall problem. *Front. Psychol.* 6:1168 10.3389/fpsyg.2015.01168 PMC4531217 26321986

[B4] Barbey A. K. Sloman S. A. (2007). Base-rate respect. From ecological rationality to dual processes. *Behav. Brain Sci.* 30 241–297. 10.1017/S0140525X07001653 17963533

[B5] Barker M. J. (2017). Connecting applied and theoretical bayesian epistemology. data relevance, pragmatics, and the legal case of sally clark. *J. Appl. Philos.* 34 242–262. 10.1111/japp.12181

[B6] Batanero C. Godino J. D. Vallecillos A. Green D. R. Holmes P. (1994). Errors and difficulties in understanding elementary statistical concepts. *Int. J. Math. Educ. Sci. Technol.* 25 527–547. 10.1080/0020739940250406

[B7] Binder K. Krauss S. Bruckmaier G. (2015). Effects of visualizing statistical information. An empirical study on tree diagrams and 2 × 2 tables. *Front. Psychol.* 6:1186 10.3389/fpsyg.2015.01186 PMC4549558 26379569

[B8] Binder K. Krauss S. Bruckmaier G. Marienhagen J. (2018). Visualizing the Bayesian 2-test case. The effect of tree diagrams on medical decision making. *PLoS One* 13:e195029 10.1371/journal.pone.0195029 PMC5871005 29584770

[B9] Böcherer-Linder K. Eichler A. (2017). The impact of visualizing nested sets. An empirical study on tree diagrams and unit squares. *Front. Psychol.* 7:2026 10.3389/fpsyg.2016.02026 PMC5226638 28123371

[B10] Böcherer-Linder K. Eichler A. (2019). How to improve performance in bayesian inference tasks: a comparison of five visualizations. *Front. Psychol.* 10:267 10.3389/fpsyg.2019.00267 PMC6401595 30873061

[B11] Brase G. L. (2008). Pictorial representations in statistical reasoning. *Appl. Cogn. Psychol.* 23 369–381. 10.1002/acp.1460

[B12] Brase G. L. (2014). The power of representation and interpretation. Doubling statistical reasoning performance with icons and frequentist interpretations of ambiguous numbers. *J. Cogn. Psychol.* 26 81–97. 10.1080/20445911.2013.861840

[B13] Bruckmaier G. Binder K. Krauss S. Kufner H.-M. (2019). An eye-tracking study of statistical reasoning with tree diagrams and 2 × 2 tables. *Front. Psychol.* 10:303 10.3389/fpsyg.2019.00632 PMC6530428 31156488

[B14] Budgett S. Pfannkuch M. (2019). “Visualizing chance: tackling conditional probability misconceptions,” in *Topics and Trends in Current Statistics Education Research: International Perspectives*, eds Burrill G. Ben-Zvi D. (Cham: Springer International Publishing), 3–25. 10.1007/978-3-030-03472-6_1

[B15] Budgett S. Pfannkuch M. Franklin C. (2016). “Building conceptual understanding of probability models: visualizing chance,” in *Annual Perspectives in Mathematics Education 2016: Mathematical Modeling and Modeling Mathematics*, eds Hirsch C. R. McDuffie A. R. (Reston, VA: Natl Coun Teachers Math), 37–49.

[B16] Chapman G. B. Liu J. (2009). Numeracy, frequency, and Bayesian reasoning. *Judgment Decision Making* 4 34–40.

[B17] Cosmides L. Tooby J. (1996). Are humans good intuitive statisticians after all? Rethinking some conclusions from the literature on judgment under uncertainty. *Cognition* 58 1–73. 10.1016/0010-0277(95)00664-8

[B18] Curcio F. R. (1989). *Developing Graph Comprehension.* Reston, VA: N.C.T.M.

[B19] Días C. Batanero C. (2009). University Students’ knowledge and Biases in conditional probability reasoning. *Int. Electr. J. Math. Educ.* 4 131–162.

[B20] Eddy D. M. (1982). “Probabilistic reasoning in clinical medicine: problems and opportunities,” in *Judgment Under Uncertainty: Heuristics and Biases*, eds Kahneman D. Slovic P. Tversky A. (New York, NY: Cambridge University Press), 249–267. 10.1017/cbo9780511809477.019

[B21] Eichler A. Böcherer-Linder K. (2018). “Categorizing errors in Bayesian situations,” in *Proceedings of the Tenth International Conference on Teaching Statistics (ICOTS10) Looking Back, Looking Forward.* Kyoto.

[B22] Ellis K. M. Cokely E. T. Ghazal S. Garcia-Retamero R. (2014). Do people understand their home HIV test results? Risk literacy and information search. *Proc. Hum. Fact. Ergon. Soc. Annu. Meet.* 58 1323–1327. 10.1177/1541931214581276

[B23] Garcia-Retamero R. Cokely E. T. Hoffrage U. (2015). Visual aids improve diagnostic inferences and metacognitive judgment calibration. *Front. Psychol.* 6:932 10.3389/fpsyg.2015.00932 PMC4504147 26236247

[B24] Garcia-Retamero R. Hoffrage U. (2013). Visual representation of statistical information improves diagnostic inferences in doctors and their patients. *Soc. Sci. Med.* 83 27–33. 10.1016/j.socscimed.2013.01.034 23465201

[B25] Gigerenzer G. Hoffrage U. (1995). How to improve Bayesian reasoning without instruction: frequency formats. *Psychol. Rev.* 102 684–704. 10.1037/0033295X.102.4.684

[B26] Goodie A. S. Fantino E. (1996). Learning to commit or avoid the base-rate error. *Nature* 380 247–249. 10.1038/380247a0 8637572

[B27] Hill R. (2004). Multiple sudden infant deaths – coincidence or beyond coincidence? *Paediatr. Perinatal Epidemiol.* 18 320–326. 10.1111/j.1365-3016.2004.00560.x 15367318

[B28] Hoffrage U. Gigerenzer G. (1998). Using natural frequencies to improve diagnostic inferences. *Acad. Med.* 73 538–540. 10.1097/00001888-199805000-00024 9609869

[B29] Hoffrage U. Hafenbrädl S. Bouquet C. (2015a). Natural frequencies facilitate diagnostic inferences of managers. *Front. Psychol.* 6:642 10.3389/fpsyg.2015.00642 PMC4475789 26157397

[B30] Hoffrage U. Krauss S. Martignon L. Gigerenzer G. (2015b). Natural frequencies improve Bayesian reasoning in simple and complex inference tasks. *Front. Psychol.* 6:1473 10.3389/fpsyg.2015.01473 PMC4604268 26528197

[B31] Hoffrage U. Lindsey S. Hertwig R. Gigerenzer G. (2000). Communicating statistical information. *Science* 290 2261–2262. 10.1126/science.290.5500.2261 11188724

[B32] Jessop A. (2018). “Bayes and the Law,” in *Let the Evidence Speak*, 1st Edn, eds Jessop A. J. Plotnikova A. (Berlin: Springer International Publishing), 189–205. 10.1007/978-3-319-71392-2_16

[B33] Johnson E. D. Tubau E. (2013). Words, numbers, & numeracy. Diminishing individual differences in Bayesian reasoning. *Learn. Individ. Differ.* 28 34–40. 10.1016/j.lindif.2013.09.004

[B34] Johnson E. D. Tubau E. (2017). Structural mapping in statistical word problems: a relational reasoning approach to Bayesian inference. *Psychonom. Bull. Rev.* 24 964–971. 10.3758/s13423-016-1159-6 27678378

[B35] Kahneman D. Slovic P. Tversky A. (eds) (1982). *Judgment under Uncertainty: Heuristics and Biases.* New York, NY: Cambridge University Press. 10.1126/science.185.4157.1124 17835457

[B36] Khan A. Breslav S. Glueck M. Hornbaek K. (2015). Benefits of visualization in the mammography problem. *Int. J. Hum. Comput. Stud.* 83 94–113. 10.1016/j.ijhcs.2015.07.001

[B37] Kooperationsgemeinschaft Mammographie (2018). *Jahresbericht Evaluation 2016: Deutsches Mammographie-Screening-Programm.* Berlin: Kooperationsgemeinschaft Mammographie.

[B38] Krauss S. Wang X. T. (2003). The psychology of the monty hall problem. Discovering psychological mechanism for solving a tenacious brain teaser. *J. Exp. Psychol. Gen.* 132 3–22. 10.1037/0096-3445.132.1.3 12656295

[B39] Lehner M. C. Reiss K. (2018). Entscheidungsstrategien an Vierfeldertafeln: eine Analyse mit Blickbewegungen. *J. Math. Didakt.* 39 147–170. 10.1007/s13138-018-0132-5

[B40] Luchins A. S. (1942). Mechanization in problem solving: the effect of Einstellung. *Psychol. Monogr.* 54 i–95. 10.1037/h0093502

[B41] McDowell M. Galesic M. Gigerenzer G. (2018). Natural frequencies do foster public understanding of medical tests. comment on Pighin, Gonzalez, Savadori and Girotto (2016). *Med. Decis. Making* 38 390–399. 10.1177/0272989X18754508 29448883

[B42] McDowell M. Jacobs P. (2017). Meta-analysis of the effect of natural frequencies on Bayesian reasoning. *Psychol. Bull.* 143 1273–1312. 10.1037/bul0000126 29048176

[B43] McKenzie C. R. M. (1994). The accuracy of intuitive judgment strategies: covariation assessment and bayesian inference. *Cogn. Psychol.* 26 209–239. 10.1006/cogp.1994.1007

[B44] Micallef L. Dragicevic P. Fekete J.-D. (2012). Assessing the effect of visualizations on Bayesian reasoning through crowdsourcing. *IEEE Trans. Vis. Comput. Graph.* 18 2536–2545. 10.1109/TVCG.2012.199 26357162

[B45] Oldford R. W. Cherry W. H. (2006). *Picturing Probability. The Poverty Of Venn Diagrams, the Richness Of Eikosograms.* Available online at: http://sas.uwaterloo.ca/~rwoldfor/papers/venn/eikosograms/paperpdf.pdf (accessed October 12, 2006).

[B46] Operskalski J. T. Barbey A. K. (2016). Risk literacy in medical decision-making. *Science* 352 413–414. 10.1126/science.aaf7966 27102467

[B47] Pfannkuch M. Budgett S. (2017). Reasoning from an Eikosogram: An exploratory study. *Int. J. Res. Undergrad. Math. Edn.* 3, 283–310. 10.1007/s40753-016-0043-0

[B48] Reani M. Davies A. Peek N. Jay C. (2018). How do people use information presentation to make decisions in Bayesian reasoning tasks? *Int. J. Hum. Comput. Stud.* 111 62–77. 10.1016/j.ijhcs.2017.11.004

[B49] Schapira M. M. Nattinger A. B. McHorney C. A. (2001). Frequency or probability? A qualitative study of risk communication formats used in health care. *Med. Decision Making* 21 459–467. 10.1177/0272989X0102100604 11760103

[B50] Schneps L. Colmez C. (2013). *Math on Trial: How Numbers Get Used and Abused in the Courtroom.* New York, NY: Basic Books.

[B51] Sedlmeier P. Gigerenzer G. (2001). Teaching Bayesian reasoning in less than two hours. *J. Exp. Psychol. Gen.* 130 380–400. 10.1037/0096-3445.130.3.380 11561916

[B52] Shaklee H. Hall L. (1983). Methods of assessing strategies for judging covariation between events. *J. Educ. Psychol.* 75 583–594. 10.1037/0022-0663.75.4.583

[B53] Siegrist M. Keller C. (2011). Natural frequencies and Bayesian reasoning. The impact of formal education and problem context. *J. Risk Res.* 14 1039–1055. 10.1080/13669877.2011.571786

[B54] Sirota M. Kostovièová L. Juanchich M. (2014). The effect of iconicity of visual displays on statistical reasoning. Evidence in favor of the null hypothesis. *Psychonom. Bull. Rev.* 21 961–968. 10.3758/s13423-013-0555-4 24307248

[B55] Sloman S. A. Over D. Slovak L. Stibel J. M. (2003). Frequency illusions and other fallacies. *Organ. Behav. Hum. Decis. Process* 91 296–309. 10.1016/S0749-5978(03)00021-9

[B56] Soto-Andrade J. (2019). “Missing the (Bayesian) wood for the trees?,” in *Actas del Tercer Congreso Internacional Virtual de Educación Estadística*, eds Contreras J. M. Gea M. M. López-Martín M. M. Molina-Portillo E..

[B57] Spiegelhalter D. Pearson M. Short I. (2011). Visualizing uncertainty about the future. *Science* 333 1393–1400. 10.1126/science.1191181 21903802

[B58] Starns J. J. Cohen A. L. Bosco C. Hirst J. (2019). A visualization technique for Bayesian reasoning. *Appl. Cogn. Psychol.* 33 234–251. 10.1002/acp.3470

[B59] Steckelberg A. Balgenorth A. Berger J. Mühlhauser I. (2004). Explaining computation of predictive values: 2 x 2 table versus frequency tree. A randomized controlled trial [ISRCTN74278823]. *BMC Med. Educ.* 4:13 10.1186/1472-6920-4-13 PMC514564 15301689

[B60] Sweller J. (2003). Evolution of human cognitive architecture. *Psychol. Learn. Motiv.* 43 215–266. 10.1016/S0079-7421(03)01015-6

[B61] Talboy A. N. Schneider S. L. (2017). Improving accuracy on Bayesian inference problems using a brief tutorial. *J. Behav. Dec. Making* 30 373–388. 10.1002/bdm.1949

[B62] Tubau E. Rodríguez-Ferreiro J. Barberia I. Colomé À (2019). From reading numbers to seeing ratios: a benefit of icons for risk comprehension. *Psychol. Res.* 83 1808–1816. 10.1007/s00426-018-1041-4 29931591

[B63] Ufer S. Lindmeier A. Reiss K. (2011). Würfel oder Kugel? Entscheidungsstrategien systematisieren und vergleichen. *Math. Lehren* 168 18–22.

[B64] Wassner C. (2004). *Förderung Bayesianischen Denkens - Kognitionspsychologische Grundlagen und didaktische Analysen.* Hildesheim: Franzbecker.

[B65] Weber P. Binder K. Krauss S. (2018). Why Can Only 24% solve bayesian reasoning problems in natural frequencies: frequency phobia in spite of probability blindness. *Front. Psychol.* 9:1833 10.3389/fpsyg.2018.01833 PMC6194348 30369891

[B66] Wu C. M. Meder B. Filimon F. Nelson J. D. (2017). Asking better questions: how presentation formats influence information search. *J. Exp. Psychol.* 43 1274–1297. 10.1037/xlm0000374 28318286

[B67] Yamagishi K. (2003). Facilitating normative judgments of conditional probability. Frequency or nested sets? *Exp. Psychol.* 50 97–106. 10.1026//1618-3169.50.2.97 12693194

[B68] Yan D. Davis G. E. (2018). The turtleback diagram for conditional probability. *OJS* 08 684–705. 10.4236/ojs.2018.84045

[B69] Zhu L. Gigerenzer G. (2006). Children can solve Bayesian problems. The role of representation in mental computation. *Cognition* 98 287–308. 10.1016/j.cognition.2004.12.003 16399266

[B70] Zikmund-Fisher B. J. Witteman H. O. Dickson M. Fuhrel-Forbis A. Kahn V. C. Exe N. L. (2014). Blocks, ovals, or people? Icon type affects risk perceptions and recall of pictographs. *Med. Decis. Making* 34 443–453. 10.1177/0272989X13511706 24246564 PMC3991751

