# Supplementary material for: A New Visualization for Probabilistic Situations Containing Two Binary Events: The Frequency Net
Source: Front Psychol. 2020 May 26;11:750. doi: 10.3389/fpsyg.2020.00750 (PMC7264419; doi:10.3389/fpsyg.2020.00750)
Supplement: Supplementary file 1 [file Image_1.pdf]

Supplementary Material

1 Supplementary Figures

Supplementary Figure 1 shows the percentages of correct inferences in the question for a conditional probability, separated for information format and visualization and separated for the two contexts (mammography problem vs. economics problem).

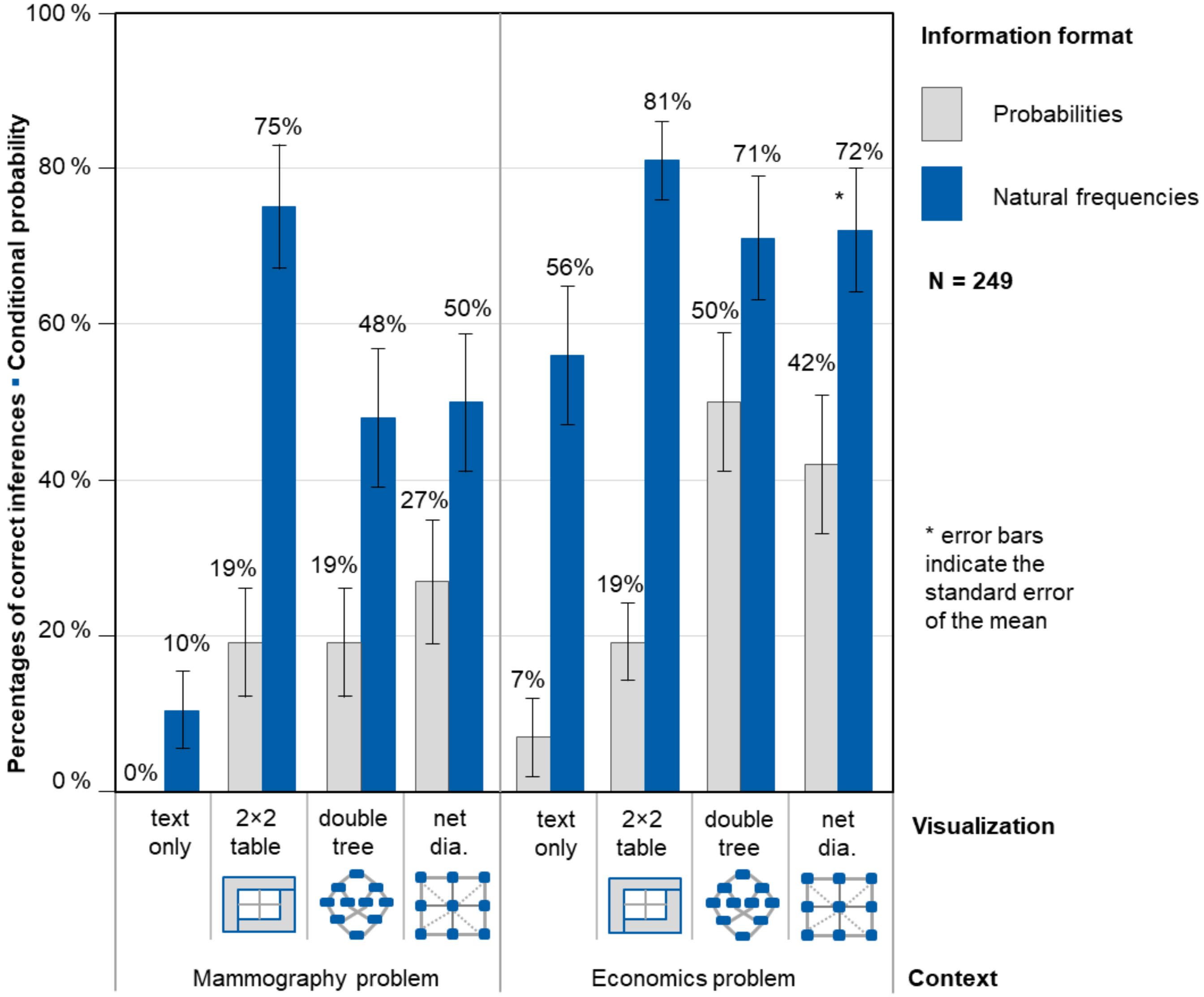

Supplementary Figure 1. Percentages of correct inferences in the question for a conditional probability, separated for information format and visualization and separated for the two contexts (mammography problem vs. economics problem)

Furthermore, supplementary Figure 2 shows the percentages of correct inferences in the question for a joint probability, separated for information format and visualization and separated for the two contexts (mammography problem vs. economics problem).

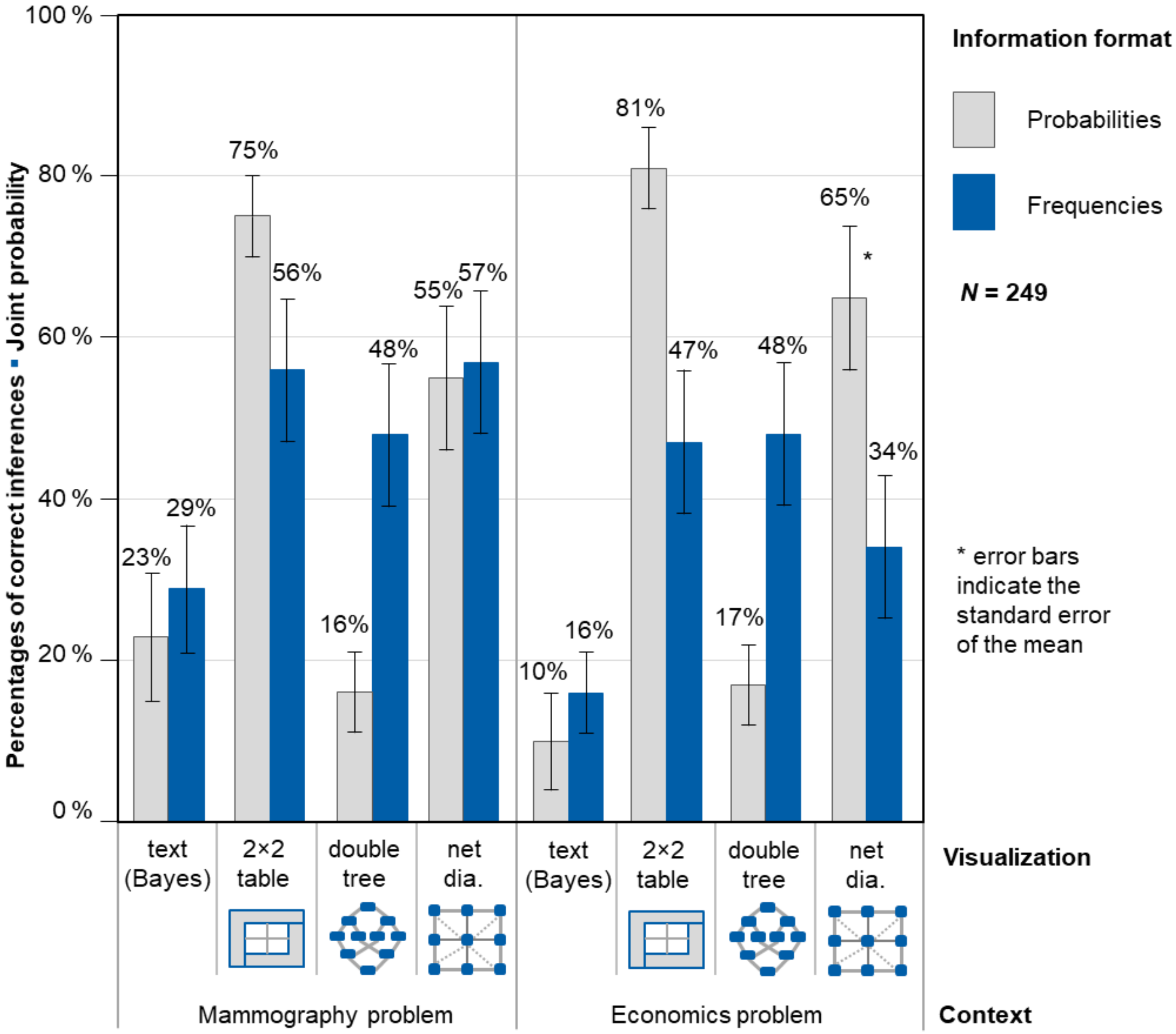

Supplementary Figure 2. Percentages of correct inferences in the question for a joint probability, separated for information format and visualization and separated for the two contexts (mammography problem vs. economics problem)
